# The American Armamentarium Chirurgicum

**Published:** 1889-06

**Authors:** 


**The American Armamentarium Chirurgicum.** George Tiemann & Co. New York. Imperial 8vo., pp. xv., 846. 1889.

Just ten years ago the well-known instrument firm of Geo. Tiemann & Co. issued a catalogue with the unique title of "The American Armamentarium Chirurgicum." It at once attracted attention as the best catalogue of instruments ever issued by a single firm. With commendable enterprise another catalogue, having the same title, is now offered to the public. It differs in many respects from the first one, and illustrates in its own way the great advance made in surgery during the last decade. It merits more attention than a simple catalogue of instruments ordinarily would receive.

In the first place, it is an attractive volume, well bound, finely printed, and filled with most excellent illustrations. There are 3,400 of the latter, and it is difficult to see how they could be improved. Each engraving gives a very clear idea of the construction of the instrument illustrated.

The distinctive feature of this catalogue, however, lies in the fact that it contains a description of the anatomy of the parts to be operated upon, as well as the method of procedure in very many of the operations. These, of course, occur under different heads as the instruments for operation on the various regions of the body are considered. The descriptions of anatomy are taken from the principal text-books, and the details of the operations from acknowledged authorities on surgical matters. As an example, we will take one of the smaller divisions, and mention the text accompanying the descriptions of the instruments.

The heading on page 240 is Tonsillar, and first is considered the Anatomy and Physiology of the Tonsils, Uvula and Soft Palate. This fills nearly two pages, and is credited to "Yearsley on the Throat." Then follows two paragraphs, one on the Nature of the Changes produced in the Tonsils and Uvula, and the other on Scarification of Enlarged Tonsils in a State of Active Inflammation. Tonsillotomy is then described, and the various methods of operation detailed, each in the words of the author proposing this or that method. Finally, a page is given to the Excision of the Uvula. All of this is accompanied by cuts of the instruments discussed for the operations.

This is the method pursued in all the different departments of surgery, and it can be seen readily that it is a most comprehensive catalogue. If it were a treatise on surgery, it would be open to the criticism that many methods of treatment were left out altogether; but this is not necessary, as from its very character it only considers the operations that may be performed by use of the instruments made by the firm issuing the catalogue.

We think the convenience of the book could be increased in one particular: the price of each instrument should follow after its name. This is done in some cases, but it should be done in all. To be sure, there is a most complete index giving name, figure, and price of all the instruments, but this necessitates finding the name again, and one is not always sure under which head to find it. We have spent some little time looking for the price of an instrument that could be indexed under several heads.

No advertisement appears in the volume, the expense being borne entirely by Messrs. Geo. Tiemann & Co.

Any physician will receive "The Armamentarium" by sending the publisher the small sum of *one dollar*, and paying either the postage or the express charges, as preferred. It seems to us that every one should take advantage of this rare opportunity and obtain this beautiful and comprehensive catalogue and price list of surgical instruments.

